# Gate-Tunable Single Terahertz Meta-Atom Ultrastrong Light-Matter Coupling

**DOI:** 10.1021/acsphotonics.5c02675

**Published:** 2026-01-28

**Authors:** Elsa Jöchl, Anna-Lydia Vieli, Lucy Hale, Felix Helmrich, Deniz Turan, Mona Jarrahi, Mattias Beck, Jérôme Faist, Giacomo Scalari

**Affiliations:** † 54177 Institute of Quantum Electronics, ETH Zürich, Zürich 8093, Switzerland; ‡ Department of Electrical & Computer Engineering, 8783 UCLA, Los Angeles, California 90095, United States

**Keywords:** ultrastrong coupling, split-ring resonator, two-dimensional electron gas, Landau levels, extreme confinement, few electrons, gate-tunability

## Abstract

We study the electrical tunability of ultrastrong light-matter interactions between a single terahertz circuit-based complementary split ring resonator (cSRR) and a two-dimensional electron gas. For this purpose, transmission spectroscopy measurements are performed under the influence of a strong magnetic field at different set points for the electric gate bias. The resulting Landau polariton dispersion depends on the applied electric bias, as the gating technique confines the electrons in-plane down to extremely subwavelength dimensions as small as *d* = 410 nm. This confinement allows for the excitation of standing plasma waves at zero magnetic field and an effective tunability of the electron number coupled to the THz resonator. This allows the normalized coupling strength to be tuned in situ from η = 0.46 down to η = 0.18. This is the first demonstration of terahertz far-field spectroscopy of an electrically tunable interaction between a single terahertz resonator and electrons in a GaAs quantum well heterostructure.

## Graphical abstract

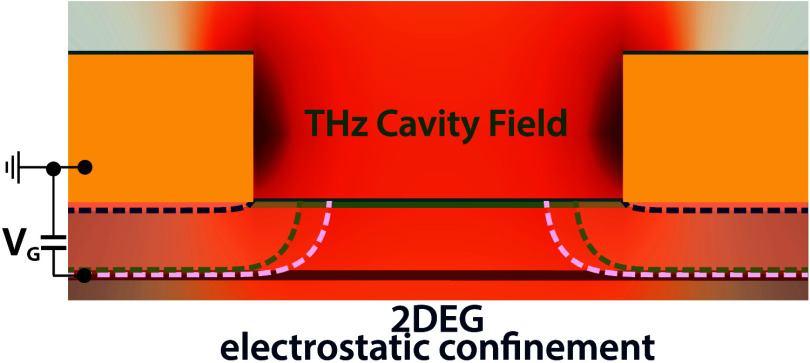

## Introduction

Ultrastrong light-matter interaction has been studied extensively in the terahertz (THz) frequency range.^1,2^ At these low energies, the Rabi-frequency of the light-matter coupled system can readily reach a significant fraction of the system’s uncoupled eigenfrequencies, which is the defining condition for ultrastrong coupling. This nonperturbative hybridization of light and matter has several fundamental implications, for example, on the ground state of the system,^3^ which hosts virtual photons. Recent experimental and theoretical efforts have highlighted the potential of such ultrastrong light-matter interactions to modify material properties.^4–9^

One way of achieving ultrastrong coupling is by confining the THz electric fields strongly in subwavelength modes through the use of circuit-based resonators, so-called split-ring resonators (SRRs).^10–14^ Furthermore, intraband transitions in semiconductor heterostructures at these frequencies can be easily integrated with these SRR modes.^15,16^ Here, we couple Landau level transitions in a GaAs single quantum well heterostructure to a single complementary SRR (cSRR) mode by applying a magnetic field perpendicular to a two-dimensional electron gas (2DEG) in a GaAs quantum well heterostructure.

There has been extensive effort in studying ultrastrong coupling with few electrons in the THz regime.^15,17–19^ In this case, the electronic excitation can no longer be treated as a harmonic, quasi-bosonic mode, and its Fermionic nature starts to play a role. The corresponding anharmonicity is predicted to result in a strikingly different polariton dispersion.^20,21^

One possibility to reduce the interaction to a small ensemble of electrons is by fabricating meta-atoms with a very low mode volume, as done in ref 17. This approach, however, does not allow for in situ tunability of the interaction. Another method is to electrically tune the electron system via a gating technique.^22^ In typical experiments measuring large (*n* > 100) arrays of collectively coupled systems, this requires uniform gating in each individual subwavelength region across a large area, which is technologically challenging. Recently, we have demonstrated far-field measurements of a single cSRR by using an asymmetric solid immersion lens (aSIL) system mounted in direct contact with the sample.^19^ In this work, we combine this single meta-atom spectroscopy technique with in situ electrical modification of the electron system below the single cSRR.

To tune the matter system, a gate bias is applied between the 2DEG and the metallic cSRR plane. By applying such a spatially inhomogeneous gate bias, the 2DEG is mainly depleted in the regions below the resonator surface, which creates a confinement in the shape of the resonator openings. Increasing the gate bias, therefore, enhances the in-plane confinement of the electron system. As a result, the overlap factor between the light and matter systems is no longer governed by the optical mode volume, but rather by the depletion-induced confinement of the electron system. This allows for dynamical tuning of the light-matter coupling strength, which depends on the number of electrons: $\hbar \Omega_{R}={\mathrm{d⃗}}_{\mathrm{ij}}\cdot {\mathrm{\varepsilon ⃗}}_{\mathrm{vac}}\sqrt{N_{e}}$ , with *N*_e_ the number of optically active electrons, *d⃗*_*ij*_ the transition dipole moment of the matter system, and ε⃗_vac_ the vacuum electric field of the resonator mode. Furthermore, shaping the 2DEG into the specific shape of the cSRR makes it possible to couple to standing plasma waves which appear at zero magnetic field and depend on the gate bias.

## Results and Discussion

### Fabrication and Methods

The sample is processed on a GaAs/AlGaAs single triangular quantum well at 90 nm depth. This structure is grown in-house using molecular beam epitaxy, and exhibits a nominal electron sheet density of *n*_e_ = 3 × 10^11^ cm^–2^ at zero electric field bias.

In order to electrically contact the quantum well, ohmic contacts are established using a standard annealing process. These contacts are established outside the allocated area of the resonator plane, approximately 2 mm away from the meta-atom. This enables an electrical contact to the 2DEG by way of wirebonding after the deposition of the conductive cSRR plane. To ensure electrical insulation of the 2DEG and avoid leakage currents through the GaAs cap layer, a thin layer of alumina (Al_2_O_3_) is deposited using atomic layer deposition. The alumina is etched down to a thickness of *t* ≤ 10 nm in the area where the cSRR will later be placed to ensure maximal overlap between the 2D electron system and the resonant cavity mode. The remaining nonzero thickness of Al_2_O_*x*_ ensures that the GaAs cap layer underneath the cSRR is protected in the later etching steps, leaving the underlying quantum well intact. Afterward, the resonator plane is deposited using a lithographic lift-off process. It consists of a circle of diameter *d* = 4 mm with a single cSRR placed in the center, and contact pads placed next to the ohmic contacts mentioned above. The cSRR is designed to be resonant at a frequency of *f* = 270 GHz. In an attempt to form a homogeneous gate, we deposited a 4 nm thin Cr layer (similarly to ref 22) to deplete the 2DEG density uniformly across the sample, according to a Schottky-contact enabled depletion mechanism. As we will show, the measurements nonetheless indicate a lateral 2DEG confinement, leading us to the conclusion that the thin Cr layer within the cSRR gap is electrically disconnected from the cSRR plane used for gating.

In the last step, a protective 2 μm thick layer of benzocyclobutene polymer (BCB) is spin coated onto the sample. As first shown in our previous work,^19^ we can utilize an aSIL system, shown in Figure 1a, to exsupercite a single meta-atom mode with far-field THz radiation, which will be coupling to our tunable 2DEG platform. The BCB-layer acts as a buffer to protect the sample from shearing damage when the lenses are put in place. This is necessary to ensure insulation between the electrically conductive layers.

**Figure 1:**
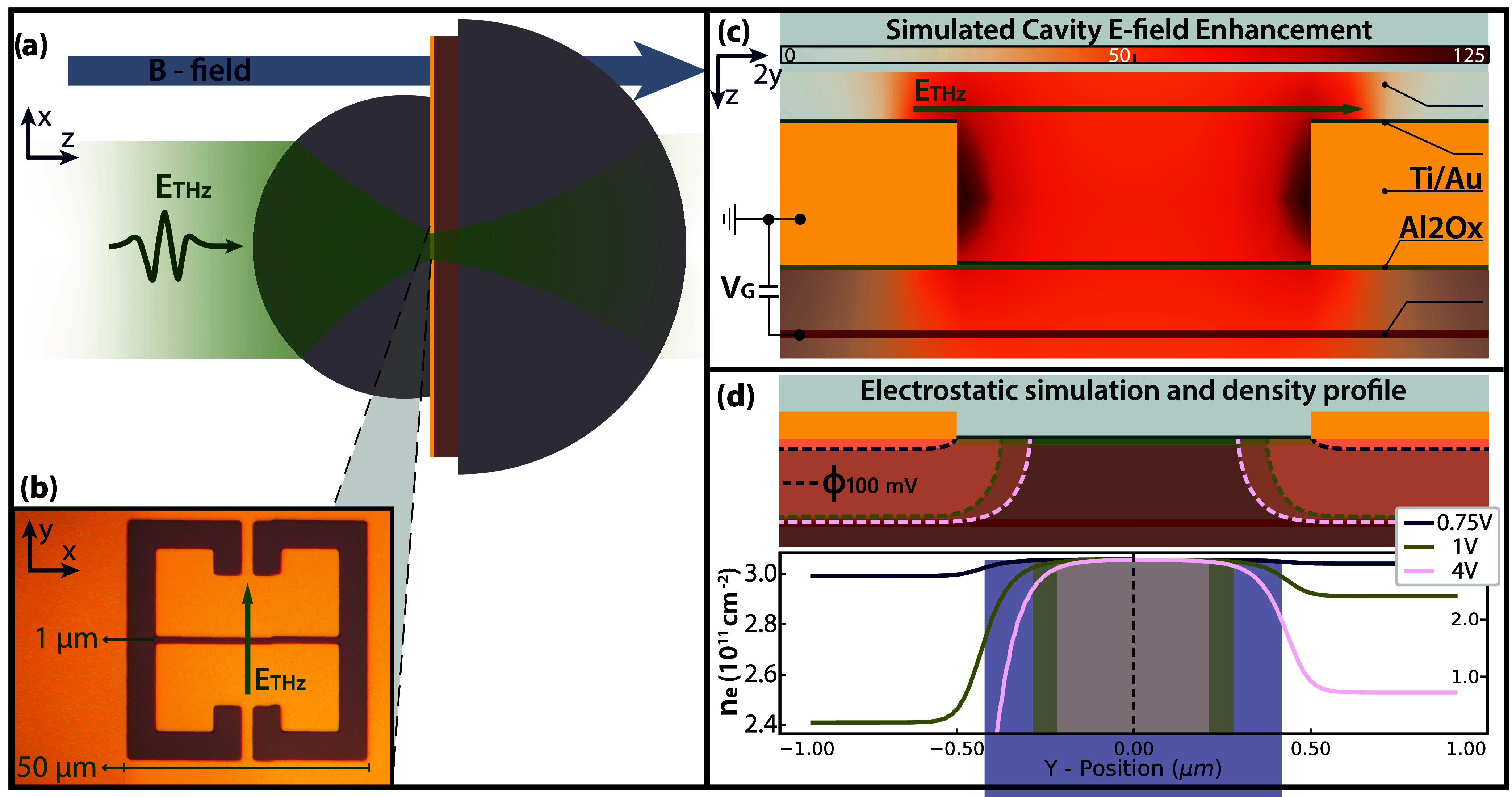
(a) Schematic of the sample mounted with aSIL system. (b) An optical image of the cSRR. (c) Schematic cut view of the sample across the cSRR gap, overlaid with the electric field confinement at zero electric bias (simulated using FEM). (d) Simulated DC field distribution (equipotential lines at Φ = 100 mV indicated with dotted lines) and lateral narrowing of the 2DEG strip due to an increase in DC bias. The aspect ratio between the *x* and *y* axis in the schematic panels of (c, d) is set to 1:2 to improve visibility of the thin layers. The computed change in the electron density for increasing potential is shown in the bottom of panel (d), according to a Thomas-Fermi distribution. To show the asymptotic values for all cases, the *y*-axis is squeezed in the right side of the plot.

After mounting the sample together with the aSILs in a cleanroom environment, it is placed in a cryomagnet system, where we perform THz transmission time-domain spectroscopy (TDS) at 3 K as a function of the magnetic field with different gate biases. The utilized THz source is presented in ref 23, and driven by a mode-locked Ti:Sapph oscillator at 800 nm. The THz field is detected by electro-optic sampling in ZnTe. The mounted aSIL structure along with the *E*-field polarization and direction of the magnetic field are indicated in Figure 1a. An optical picture of the fabricated cSRR can be seen in Figure 1b, along with a schematic side cut view along the cSRR gap in Figure 1c, where the simulated electric field confinement is overlaid.

### Results

The measured spectrum for zero electric bias is shown in Figure 2. The transmission map shows the polariton dispersion as a function of magnetic field, spanning from −3 to 3 T. The spectral map is extended to negative magnetic fields to better visualize the features appearing at *B* = 0 T. The lower polariton (LP) branches are symmetric in *B*, well-defined, and bend from 150 GHz up to the cavity frequency *f* = 255 GHz around the anticrossing at |*B*| = 0.7 T. The upper polariton (UP) appears as a broadband transmission region between 300 and 450 GHz at magnetic fields below |*B*| = 1 T. The transmission peak appearing below the slope of the cyclotron resonance upward of *f* = 400 GHz is an artifact of the normalization procedure, and is not of interest for the further analysis.

**Figure 2:**
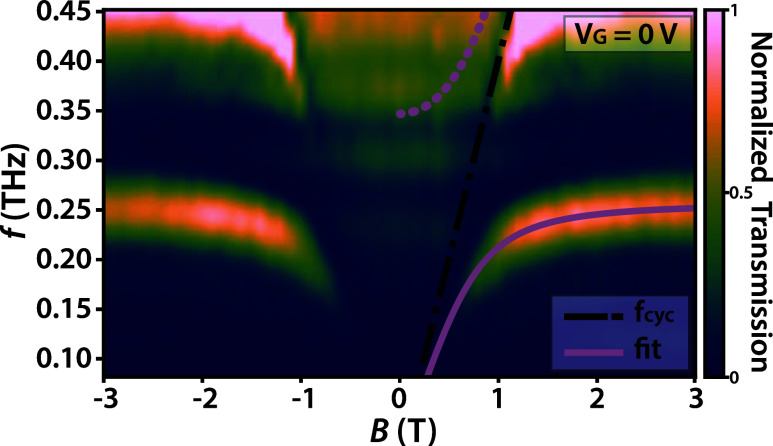
THz TDS measurements of the single cSRR sample shown in Figure 1, performed at 3 K without gate bias. The solid purple curve represents the fitted lower polariton branch according to the Hopfield model, while the dotted line represents the expected upper polariton dispersion. We observe broadened transmission instead of a localized branch due to plasmonic broadening. The black line shows the bare cyclotron dispersion corresponding to an effective mass of *m*_eff_ = 0.07 *m*_e_.

In order to understand the measured spectra, we can compare the measured data to the expected Hopfield-like dispersion for a conventional ultrastrong coupling interaction between Landau-quantized electrons and a cavity mode. We expect two polariton branches with frequencies given by^24^

1 $$\omega_{\mathrm{LP}}^{\mathrm{UP}}=\sqrt{\frac{1}{2}\left(w_{\mathrm{cyc}}^{2}+4\Omega_{R}^{2}+\omega_{\mathrm{cav}}^{2}\pm G\right)}$$

with the polariton gap *G*

2 $$G=\sqrt{-4\omega_{\mathrm{cyc}}^{2}\omega_{\mathrm{cav}}^{2}+{\left(-\omega_{\mathrm{cyc}}^{2}-4\Omega_{R}^{2}-\omega_{\mathrm{cav}}^{2}\right)}^{2}}$$

Here, ω_cyc_ denotes the bare cyclotron transition, ω_cav_ the cSRR mode, and Ω_R_ the vacuum Rabi frequency.

While the LP branches follow this expected dispersion behavior, the UP is affected by polaritonic nonlocality and does not exhibit a single well-defined frequency.^25,26^ The dispersion of the UP broadens because the electrons couple to a continuum of plasma excitations with different in-plane momenta made accessible via the fundamental cSRR mode confined in the gap of size *W* = 1 μm. As the gap size decreases, the highest possible photonic momentum mode *k* ∝ 1/*W* also increases. The upper bound for the continuum is given by the magnetoplasmon frequency

3 $$\omega_{\mathrm{MP}}=\sqrt{\frac{n_{e}e^{2}\pi }{2m^{*}\epsilon_{\mathrm{eff}}W}}$$

with *n*_e_ the electron sheet density, *e* the electron charge, m* the electron effective mass, and ϵ_eff_ the effective dielectric constant of the medium. If the UP appears below this frequency, it couples to the continuum and broadens. For a resonator with a gap of 1 μm coupling to a 2DEG of sheet density *n*_e_ = 3 × 10^11^ cm^–2^, this magnetoplasmon continuum has an upper limit of *f* = 650 GHz, well above the expected UP frequency. A UP with frequencies below this limit will therefore appear as smeared out, as is reflected in the measurement.

The Hopfield model provides an expectation of the LP given by eq 1. By fitting the measured LP data to this curve, we can quantify the normalized coupling ratio to be η = Ω/ω = 0.456 with a cavity frequency of 255 GHz.

After the calibration measurement at *V*_G_ = 0, we can study how an applied gate bias modifies the light-matter interaction. In Figure 3a–c we present measurements of the same sample at increasing gate biases. These measurements were performed in separate magnetic field sweeps. To account for small changes in the optical alignment, we performed calibration measurements at zero magnetic field and gate bias before each scan. Starting at a gate bias of *V*_G_ = 0.75 V, we observe a strong modification of the LP dispersion. First, we observe that the LP branch blue-shifts in frequency below the anticrossing, with a nonzero asymptotic value at *B* = 0 T. This asymptotic value furthermore blue shifts from 180 GHz up to 200 GHz as the voltage bias is increased to 4 V. Second, a transmission maximum forms slightly above the LP asymptotic value, which peaks at *f* = 225 GHz at the lowest gate bias, and blueshifts with increasing bias up to *f* = 280 GHz. This additional feature is indicated by a white dotted line in Figure 3a, and will furthermore be referred to as the M1 mode. The effects of an inverted gate bias on the interaction have been studied by way of finite-element simulations. The results, provided in the Supporting Information, suggest a 5% increase in coupling strength, as well as a 25 GHz red-shift of the cavity mode for a density increased by a factor of 1.5 under the cSRR.

**Figure 3:**
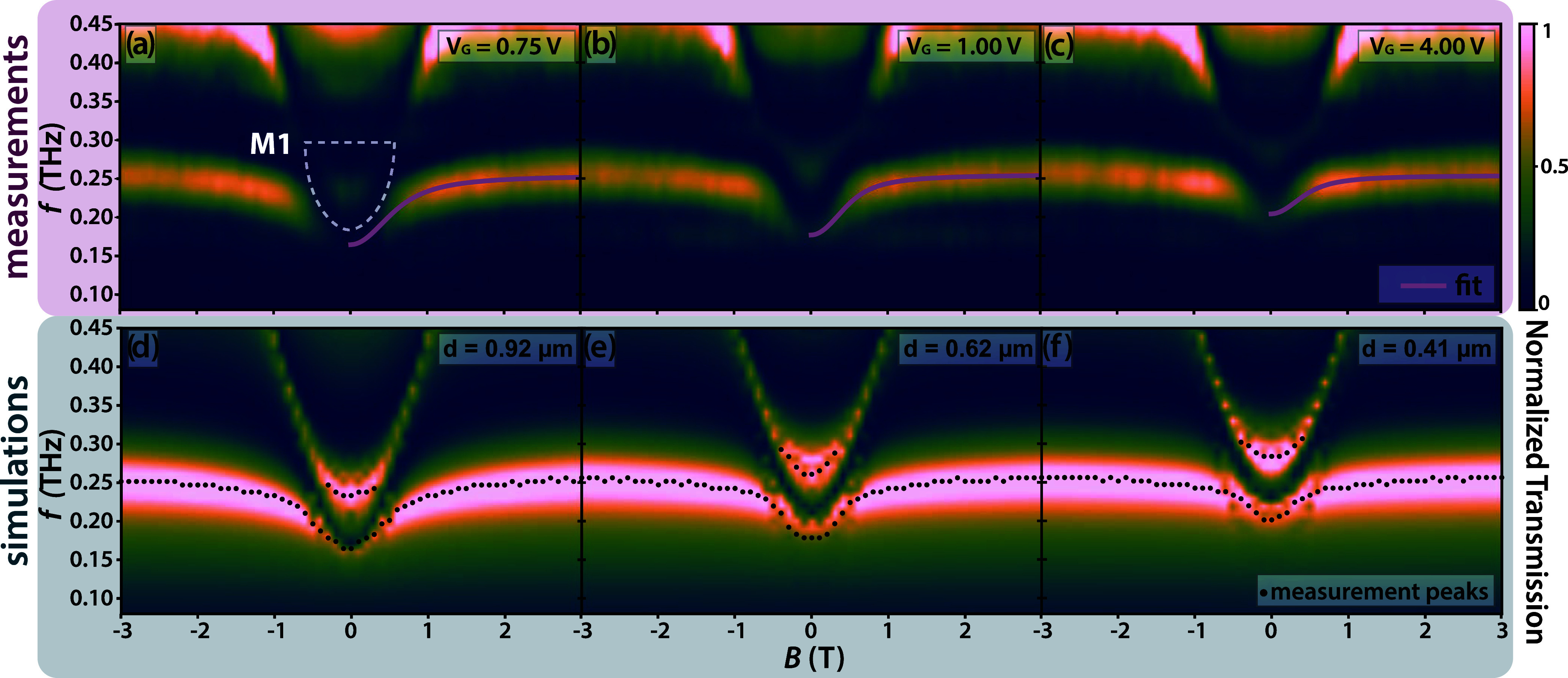
(a–c) THz TDS measurements of the single cSRR shown in Figure 1, performed at 3 K for varying back-gate biases. The purple curve represents the fitted lower polariton branch according to the Hopfield model (eq 1). As discussed above, we do not expect to observe an upper polariton branch due to plasmonic broadening, hence why the UP fit curves are omitted. Subset (a) includes a white dashed line, outlining the M1 mode. (d–f) Simulations of the sample with varying depletion lengths, overlaid with the transmission peaks extracted from the measured data for comparison.

To gain insights about the coupling strength at varying gate biases, the Hopfield model can be used to fit the LP branches. However, in this case, the cyclotron frequency ω_cyc_ in eq 1 is renormalized by the magnetoplasmon frequency given in eq 3, to account for the nonzero asymptote of the LP branches at *B* = 0 T, as done in ref 22, ${\mathrm{\omega ̃}}_{\mathrm{cyc}}=\sqrt{\omega_{\mathrm{cyc}}^{2}+\omega_{\mathrm{MP}}^{2}}$ . The normalized coupling ratios estimated with this method are decreasing with increasing gate bias η_0.75V_ = 0.33, η_1V_ = 0.23, and η_4V_ = 0.18, with the fitted magnetoplasmon frequencies *f*_0.75V_ = 218 GHz, *f*_1V_ = 210 GHz and *f*_4V_ = 240 GHz.

The emerging M1 mode is not trivially explained. It cannot originate from the UP branch, since its center frequency lies below the cavity frequency at the lowest applied gate bias *V*_G_ = 0.75 V. Furthermore, the excitation blue-shifts with increasing bias. As the coupling strength decreases with increasing bias, we would expect a UP branch to red-shift. The M1 mode, in fact, stems from the way the gate bias is applied to the 2DEG. As the voltage bias is applied directly using the resonator plane, the cSRR imprints its shape onto the 2DEG, which confines the electron system laterally. As a result, the higher the applied DC bias, the more tightly the 2DEG will be confined in-plane. Finite-element simulations performed in COMSOL support this intuitive picture.

First, we can investigate the 2D electrostatic distribution of the electric potential along a sidecut of the cSRR gap with increasing applied potentials. The results of such a simulation are shown in Figure 1d, where the respective equipotential lines at 100 mV are indicated, representing the cutoff of electric potential for different voltage biases. From this, we can calculate the electron density *n*_e_ along the *z*-position of the 2DEG according to a Thomas-Fermi distribution.^27^ The density starts to decrease by 1% at the position where the electric potential drops, which generates an effective electron channel of width *d*, confined beneath the cSRR gap. This channel varies in width from roughly 90 μm down to 40 μm at *V*_G_ = 4 V. The exact simulation parameters and potential distributions are detailed in the Supporting Information.

Then, the polariton dispersion is simulated with varying confinement strengths, corresponding to the obtained estimated effective widths *d* of the electrostatic simulation. The simulation model accounts for a 2DEG (modeled as a gyrotropic medium) with an abrupt cutoff in the shape of the cSRR, placed underneath the actual metallic cSRR. The width of this cSRR-shaped 2DEG is varied to model the effective confinement width of the 2DEG in the central cSRR gap. The simulated magnetic field is then swept to tune the cyclotron dispersion and replicate the measured transmission maps as closely as possible. The optimal simulated spectral maps correspond to channel widths *d*_0.75V_ = 0.92 μm, *d*_1V_ = 0.62 μm, and *d*_4V_ = 0.41 μm, and are shown in Figure 3d–f. These spectra show good agreement with the measurements as well as the expected widths from the electrostatic simulations for the corresponding bias voltages. The transmission peaks extracted from the measurements are overlaid with the simulated data for direct comparison. We can therefore conclude that the emerging spectral feature stems from the inhomogeneous confinement of the 2DEG.

By constraining the 2DEG spatially in this way, standing plasma wave excitations are induced within the 2DEG, akin to those reported in previous studies on geometrically patterned and gate-tunable semiconductor 2DEGs.^28–31^ These plasma waves hybridize with the LP branch. An example simulation of such a standing wave pattern is shown in Figure 4a–c. In this case, only the laterally confined 2DEG is simulated, and the cSRR is omitted to investigate the plasma excitations irrespective of their hybridization with a cavity mode.

**Figure 4:**
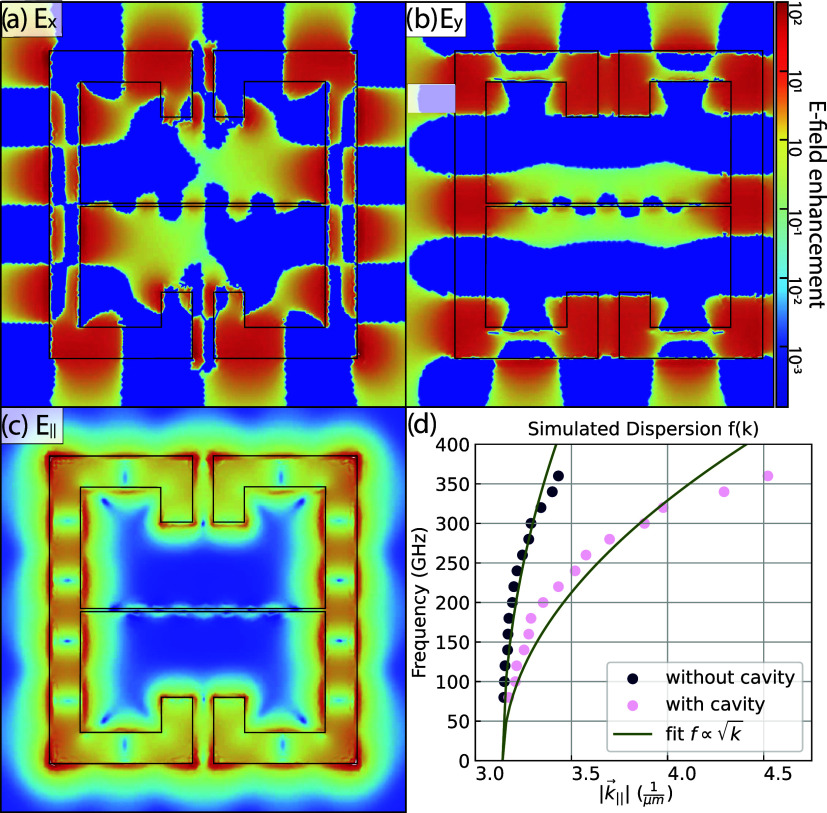
Plasma excitations of the bare 2DEG. (a–c) Normalized *x*-, *y*- and in-plane component of the simulated electric field for a 2DEG confined to the cSRR geometry without the resonator plane. The cutoff of the cSRR-shaped 2DEG is depicted with a black line. Represented at 200 GHz and a confinement width of *d* = 0.41 μm in the central gap. (d) The resulting dependence of the frequency on the in-plane wavevector, as inferred from the field distribution. Overlaid with a square-root fit for comparison.

The *E*_*x*_ and *E*_*y*_ components of the in-plane electric field (normalized to the input electric field) are plotted in Figure 4a,b respectively, and exhibit a clear standing wave pattern. The full in-plane electric field distribution is shown in Figure 4c. The plasma excitations exhibit the same dependence on the wave vector as the magnetoplasmon described in eq 3, namely ω ∝ √*k*, as established by estimating their wavelengths from the simulated in-plane electric field distributions at different frequencies. The resulting values are reported in Figure 4d.

Finally, we can estimate the number of ultrastrongly coupled electrons and compare the finite-bias cases with the nominal measurement at 0 V bias. At zero bias, the number of interacting electrons is given by^17^

4 $$N_{e}^{0}=\frac{\mathrm{eB}}{h}\times S_{\mathrm{eff}}\approx 6510$$

where *S*_eff_ = 41 μm^2^ is the effective cavity surface at the position of the electron system, computed analytically with finite-element simulations. The magnetic field *B* = 0.66 T is the field where a cyclotron mode with effective mass 0.07 *m*_e_ crosses with a cavity mode at 263 GHz. This mode frequency is taken to be in between the empty cavity mode, and the LP asymptote obtained from the fit of the coupled system. A detailed estimation of the uncertainty on the electron numbers (see the Supporting Information) yields a maximum of 10% for both methods we are about to present.

Equation 4 for the electron number becomes inaccurate when a magnetoplasmon continuum forms, as the Landau Level quantization of the electrons is now modified by nonlocal effects.^17^ However, we can relate the Rabi frequency to the number of electrons $\Omega_{R}\propto \sqrt{N_{e}}$ , and take the ratio of the coupling strength at different gate bias setting. This procedure yields a minimal electron number of $N_{e}={\left(\frac{\eta_{4V}}{\eta_{0}}\right)}^{2}\times N_{e}^{0}=1040$ at 4 V gate bias. Exact numbers for all measurements are given in Table 1.

**Table 1:** Summary of the Applied Gate Biases, Simulated Electron Channel Widths, Coupling Strengths Obtained from Hopfield Fits, the Coupling Strength Relative to the Zero Gate Bias Measurement, the Electron Number *N*_e_ Computed via Those Ratios, the Overlap Factors, and Electron Numbers Obtained via the Overlap Factors *N*_e_^*^

bias (V)	width *d* (μm)	η	(η/η_0_)^2^	Γ_V_	*N*_e_ (*N*_e_^0^ × (η/η_0_)^2^)	*N*_e_^*^ (*N*_0_ × Γ_V_)
0	-	0.46	1	1	6510	6510
0.75	0.92	0.33	0.52	0.55	3390	3580
1.00	0.62	0.23	0.26	0.31	1690	2020
4.00	0.41	0.18	0.16	0.18	1040	1170

To further verify the decrease in the fitted coupling strengths and the subsequently obtained decrease in the electron number *N*_e_, the light-matter coupling can be studied in terms of the overlap factor

5 $$\Gamma_{V}=\frac{1}{\Gamma_{0}}\frac{\int_{S_{V}}\left|E_{\mathrm{xy}}\right|dS}{\int_{S}\left|E_{\mathrm{xy}}\right|dS}$$

It is defined as the ratio of the absolute in-plane electric field |*E*_*xy*_| within the surface of the confined 2DEG channel

$$S_{V}=\int dx{\int_{-d/2}^{d/2}dy\left|E_{\mathrm{xy}}\right||}_{z=z2\mathrm{DEG}}$$

normalized to the entire electric field in the 2DEG plane (over the surface *S* extending beyond the 2DEG constraints). The normalization factor Γ_0_ is chosen so that the overlap factor is equal to unity in the case of no electron confinement. Finite element simulations provide the electric field values to compute the overlap factor for the gate biases applied in the measurements. The resulting overlap factors are reported in Table 1. Since the coupling strength depends on the magnitude of the electric field, the number of ultrastrongly coupled electrons scales in the same fashion as Γ_V_, which shows good agreement with the analysis performed via the coupling strengths, and yields a minimal reached electron number of *N*_e_^*^ = 1170.

Electrical gating, therefore, provides a method to tune the number of ultrastrongly coupled electrons by almost an order of magnitude. The limitation is currently given by leakage currents breaking through the insulating layer of alumina. Spatially confining the electron system through etching, and combining such a structure with a cSRR with a lower mode volume as done in ref 15,17 , would solve these limitations. In such a resonator, the electron number could be decreased down to *N*_e_ < 10 by gate-tuning the light-matter coupling strength to η = 0.1 with the presented method.

## Conclusion

We have successfully performed in situ modification of the ultrastrong light-matter interaction between a single THz meta atom mode and a 2DEG, tuning the electron number by almost an order of magnitude. We have shown that spectroscopy on these tunable systems is possible by way of an aSIL system combined with an optimized fabrication process. The observed polariton dispersion is strongly modified by the application of an inhomogeneous voltage bias, which induces standing plasma waves in the two-dimensional electron system. These standing waves have been examined at different confinement widths and frequencies, and show a dispersion relation similar to conventional plasma excitations in 2D electron systems. The number of ultrastrongly coupled electrons and the coupling strength decrease as a function of increasing gate bias, which is corroborated by the study of the overlap between the cavity mode and the confined electronic mode.

This experiment constitutes a stepping stone for further experiments utilizing single-resonator THz spectroscopy with gate-tunable devices. Preemptively shaping underlying semiconductor-based electron systems into Hall Bars would assist in characterizing the electron density via electronic transport. This can further facilitate the application of a homogeneous gate bias to uniformly deplete the electron system. Being able to modify matter systems via an electrical gate at deeply subwavelength dimensions opens up the possibility to perform ultrastrong coupling experiments in a variety of unexplored platforms such as van der Waals heterostructures, as has recently been shown for bilayer graphene.^32^ There is particular interest in monolayer graphene in a magnetic field, ultrastrongly coupled to a THz cavity mode. In this system, the emergence of a superradiant phase transition has been a long-standing subject of debate.^33,34^

## Supplementary Material



## Notes

Optical image of the mounted and wirebonded sample, data fitting procedure for the LP with the Hopfield model at zero gate bias, 2D map of the electrostatic simulations for varying widths, details and results of the simulations of the transmission spectra, simulated spectrum of a doping scheme, utilized simulation results to calculate the overlap factors, explanation of error propagation in electron number calculations (PDF)

## References

[1] Kockum A. F., Miranowicz A., De Liberato S., Savasta S., Nori F. (2019). Ultrastrong
coupling between light and matter. Nat. Rev.
Phys..

[2] Forn-Díaz P., Lamata L., Rico E., Kono J., Solano E. (2019). Ultrastrong
coupling regimes of light-matter interaction. Rev. Mod. Phys..

[3] Ciuti C., Bastard G., Carusotto I. (2005). Quantum vacuum
properties of the
intersubband cavity polariton field. Phys. Rev.
B.

[4] Lu I.-T., Shin D., Svendsen M. K., Latini S., Hübener H., Ruggenthaler M., Rubio A. (2025). Cavity engineering of solid-state
materials without external driving. Adv. Opt.
Photonics.

[5] Anappara A. A., de Liberato S., Tredicucci A., Ciuti C., Biasiol G., Sorba L., Beltram F. (2009). Signatures of the ultrastrong light-matter
coupling regime. Phys. Rev. B.

[6] Appugliese F., Enkner J., Paravicini-Bagliani G.
L., Beck M., Reichl C., Wegscheider W., Scalari G., Ciuti C., Faist J. (2022). Breakdown of topological protection by cavity vacuum fields in the
integer quantum Hall effect. Science.

[7] Enkner J., Graziotto L., Boriçi D., Appugliese F., Reichl C., Scalari G., Regnault N., Wegscheider W., Ciuti C., Faist J. (2025). Tunable vacuum-field
control of fractional
and integer quantum Hall phases. Nature.

[8] Kim D., Dasgupta S., Ma X. (2025). Observation of the magnonic
Dicke superradiant phase transition. Sci. Adv..

[9] Ashida Y., İmamoğlu A., Faist J., Jaksch D., Cavalleri A., Demler E. (2020). Quantum Electrodynamic
Control of
Matter: Cavity-Enhanced Ferroelectric Phase Transition. Phys. Rev. X.

[10] Pendry J., Holden A., Robbins D., Stewart W. (1999). Magnetism
from conductors
and enhanced nonlinear phenomena. IEEE Trans.
Microwave Theory Tech..

[11] Scalari G., Maissen C., Hagenmüller D., De Liberato S., Ciuti C., Reichl C., Wegscheider W., Schuh D., Beck M., Faist J. (2013). Ultrastrong light-matter
coupling at terahertz frequencies with split ring resonators and inter-Landau
level transitions. J. Appl. Phys..

[12] Bayer A., Pozimski M., Schambeck S., Schuh D., Huber R., Bougeard D., Lange C. (2017). Terahertz Light–Matter Interaction
beyond Unity Coupling Strength. Nano Lett..

[13] Mornhinweg J., Diebel L. K., Halbhuber M., Prager M., Riepl J., Inzenhofer T., Bougeard D., Huber R., Lange C. (2024). Mode-multiplexing
deep-strong light-matter coupling. Nat. Commun..

[14] Halbhuber M., Mornhinweg J., Zeller V., Ciuti C., Bougeard D., Huber R., Lange C. (2020). Non-adiabatic stripping of a cavity
field from electrons in the deep-strong coupling regime. Nat. Photonics.

[15] Jeannin M., Mariotti Nesurini G., Suffit S., Gacemi D., Vasanelli A., Li L., Davies A. G., Linfield E., Sirtori C., Todorov Y. (2019). Ultrastrong
Light–Matter Coupling in Deeply Subwavelength THz LC Resonators. ACS Photonics.

[16] Todorov Y., Andrews A. M., Colombelli R. (2010). Ultrastrong Light-Matter
Coupling Regime with Polariton Dots. Phys. Rev.
Lett..

[17] Keller J., Scalari G., Cibella S., Maissen C., Appugliese F., Giovine E., Leoni R., Beck M., Faist J. (2017). Few-Electron
Ultrastrong Light-Matter Coupling at 300 GHz with Nanogap Hybrid LC
Microcavities. Nano Lett..

[18] Kuroyama K., Kwoen J., Arakawa Y., Hirakawa K. (2024). Coherent Interaction
of a Few-Electron Quantum Dot with a Terahertz Optical Resonator. Phys. Rev. Lett..

[19] Rajabali S., Markmann S., Jöchl E., Beck M., Lehner C. A., Wegscheider W., Faist J., Scalari G. (2022). An ultrastrongly coupled
single terahertz meta-atom. Nat. Commun..

[20] Todorov Y., Sirtori C. (2014). Few-Electron Ultrastrong
Light-Matter Coupling in a
Quantum LC Circuit. Phys. Rev. X.

[21] Casanova J., Romero G., Lizuain I. (2010). Deep Strong Coupling
Regime of the Jaynes-Cummings Model. Phys. Rev.
Lett..

[22] Paravicini-Bagliani G.
L., Scalari G., Valmorra F., Keller J., Maissen C., Beck M., Faist J. (2017). Gate and magnetic field tunable ultrastrong
coupling between a magnetoplasmon and the optical mode of an LC cavity. Phys. Rev. B.

[23] Turan D., Corzo-Garcia S. C., Yardimci N. T., Castro-Camus E., Jarrahi M. (2017). Impact of the Metal Adhesion Layer on the Radiation
Power of Plasmonic Photoconductive Terahertz Sources. J. Infrared, Millimeter, Terahertz Waves.

[24] Hagenmüller D., De Liberato S., Ciuti C. (2010). Ultrastrong coupling between a cavity
resonator and the cyclotron transition of a two-dimensional electron
gas in the case of an integer filling factor. Phys. Rev. B.

[25] Rajabali S., Cortese E., Beck M., De Liberato S., Faist J., Scalari G. (2021). Polaritonic nonlocality in light–matter
interaction. Nat. Photonics.

[26] Endo, S. R. ; Kim, D. ; Liang, S. ; Lee, G. ; Kim, S. ; Covarrubias-Morales, A. ; Seo, M. ; Manfra, M. J. ; Lee, D. ; Bamba, M. ; Kono, J. Cavity-Mediated Coupling between Local and Nonlocal Modes in Landau Polaritons, arXiv:2509.05738. arXiv.org e-Print archive, 2025 https://arxiv.org/abs/2509.05738. 10.1515/nanoph-2025-0442 PMC12714039 41424882

[27] Beenakker, C. W. J. ; van Houten, H. Solid State Physics; Ehrenreich, H. ; Turnbull, D. , Eds.; Academic Press, 1991; Vol. 44, pp 1–228.

[28] Dyakonov M., Shur M. (1993). Shallow water analogy for a ballistic field effect transistor: New
mechanism of plasma wave generation by dc current. Phys. Rev. Lett..

[29] Shaner E. A., Lee M., Wanke M. C., Grine A. D., Reno J. L., Allen S. J. (2005). Single-quantum-well
grating-gated terahertz plasmon detectors. Appl.
Phys. Lett..

[30] Otsuji T., Shur M. (2014). Terahertz Plasmonics:
Good Results and Great Expectations. IEEE Microwave
Mag..

[31] Sai P., Korotyeyev V. V., Dub M., Słowikowski M., Filipiak M., But D. B., Ivonyak Y., Sakowicz M., Lyaschuk Y. M., Kukhtaruk S. M., Cywiński G., Knap W. (2023). Electrical Tuning of Terahertz Plasmonic
Crystal Phases. Phys. Rev. X.

[32] Helmrich, F. ; Adlong, H. S. ; Khanonkin, I. ; Kroner, M. ; Scalari, G. ; Faist, J. ; Imamoglu, A. ; Nova, T. F. Cavity-Driven Attractive Interactions in Quantum Materials, arXiv:2408.00189. arXiv.org e-Print archive, 2025 https://arxiv.org/abs/2408.00189. 10.1038/s41586-026-10609-1 PMC13661254 42203865

[33] Hagenmüller D., Ciuti C. (2012). Cavity QED of the Graphene
Cyclotron Transition. Phys. Rev. Lett..

[34] Chirolli L., Polini M., Giovannetti V., MacDonald A. H. (2012). Drude Weight,
Cyclotron Resonance, and the Dicke Model of Graphene Cavity QED. Phys. Rev. Lett..

