# Supplementary material for: Gate-Tunable Single Terahertz Meta-Atom Ultrastrong Light-Matter Coupling
Source: ACS Photonics. 2026 Jan 28;13(4):1122–8. doi: 10.1021/acsphotonics.5c02675 (PMC12922476; doi:10.1021/acsphotonics.5c02675)
Supplement: Supplementary file 1 [file ph5c02675_si_001.pdf]

Supporting Information
Gate-tunable single terahertz meta-atom ultrastrong light-matter coupling

Elsa Jöchl*[1], Anna-Lydia Vieli[1], Lucy Hale[1], Felix Helmrich[1], Deniz Turan[2], Mona Jarrahi[2], Mattias Beck[1], Jérôme Faist[1], and Giacomo Scalari[1]

[1]Institute of Quantum Electronics, ETH Zürich, Zürich 8093, Switzerland
[2]Department of Electrical & Computer Engineering, UCLA, Los Angeles 90095, CA, USA

*Email: ejoechl@phys.ethz.ch

Supporting Information consisting of pages S1-S10 and Figures S1-S8.

Sample Mounting

The sample is mounted on an aluminium holder that allows to mount the asymmetric Si lens system on top of the sample, with an opening on one side to allow for wirebonds between the sample and printed circuit board (PCB), as shown in Figure S1. The smaller Si lens was placed on top of the sample surface after the picture was taken.

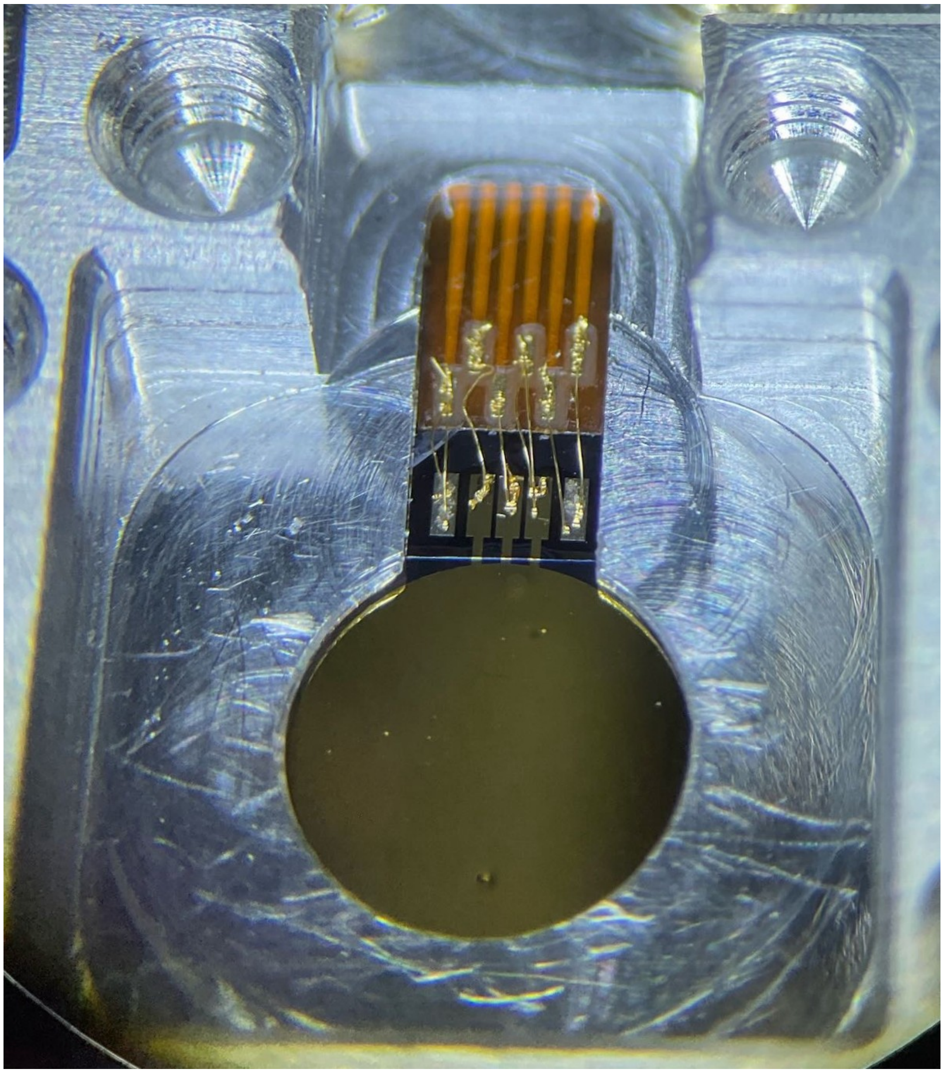

Figure S1: Optical Image of the mounted sample with the PCB and wirebonds showing at the top of the image. The large Au circle is the same size as the smaller Si lens to facilitate alignment, as well as shielding THz radiation from passing around the cSRR plane into the detector. The cSRR (not visible in this image) is situated at the center of this circle.

Data Fitting

To fit the data according to the Hopfield model, as shown in Figures 2 and 3 of the main text, the lower polariton branch peaks were extracted from the measurements as shown in S2. First, a frequency range is defined, within which the polariton branches are centered. Then, the considered spectrum transmission peaks are reported and fed into the model, wherein the cavity frequency (and for the gated measurements also the plasma frequency) are swept over a preselected frequency range. Then, the fitted LP branch is calculated and

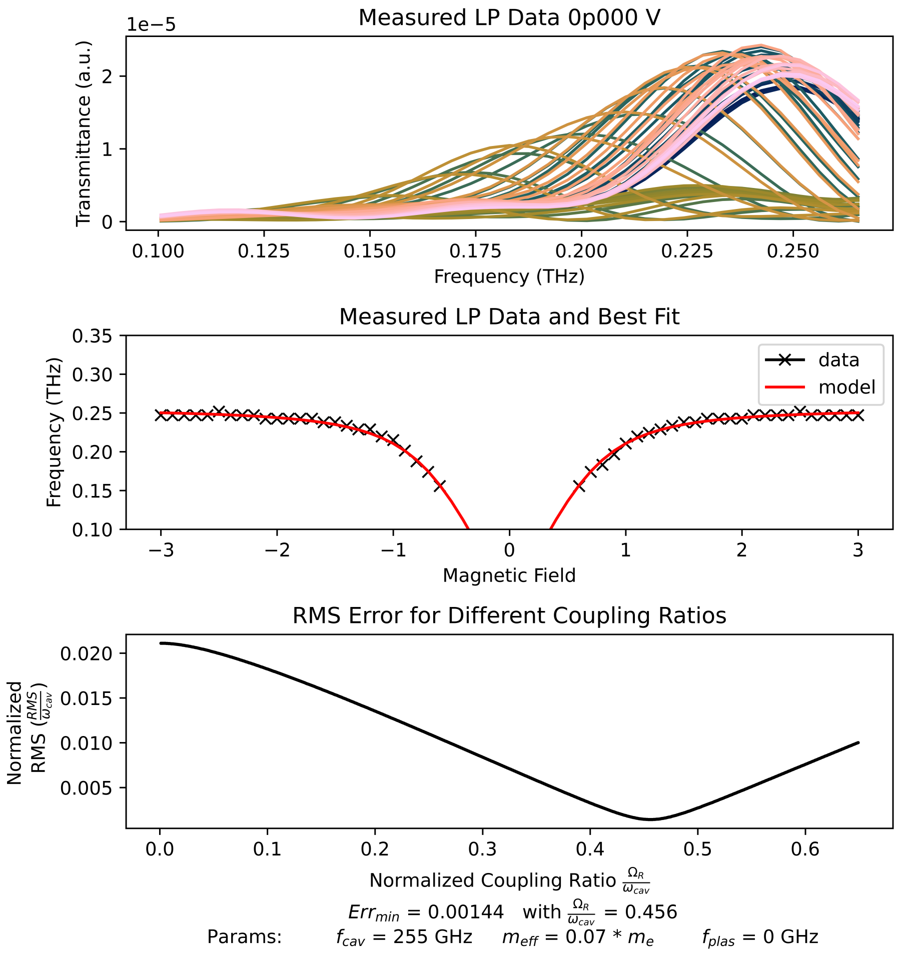

Figure S2: Fitting method to find the Hopfield Model parameters with the minimum RMS error. On top: The considered spectra, Middle: The extracted peaks from those spectra, overlaid with the optimal found fit. Bottom: RMS error as a function of the fitted coupling strength.

the RMS error is estimated for different coupling strengths. In this way, we can optimize the fit parameters dynamically, while estimating which coupling strength best represents the observed interaction. This is repeated for every measurement map to retrieve the values reported in the manuscript.

Finite Element Simulations

All simulations included in the main text of the article have been performed with the finite element simulation software COMSOL Multiphysics (version 6.4).

Electrostatic Simulation

To simulate how the 2DEG is affected by an inhomogeneous gate bias, the electrostatic module of COMSOL Multiphysics was used. A 2D simulation of a slab of GaAs containing a surface charge at the 2DEG position at $z = 0.5\,\mu$m with varied gate biases is shown in Figure S3. The gate biases do not exactly correspond to the realistic electrical potential values, and were used as parameters to replicate the simulated electron channel width (obtained from the simulation spectra). This is justified by considering that the realistic potential will

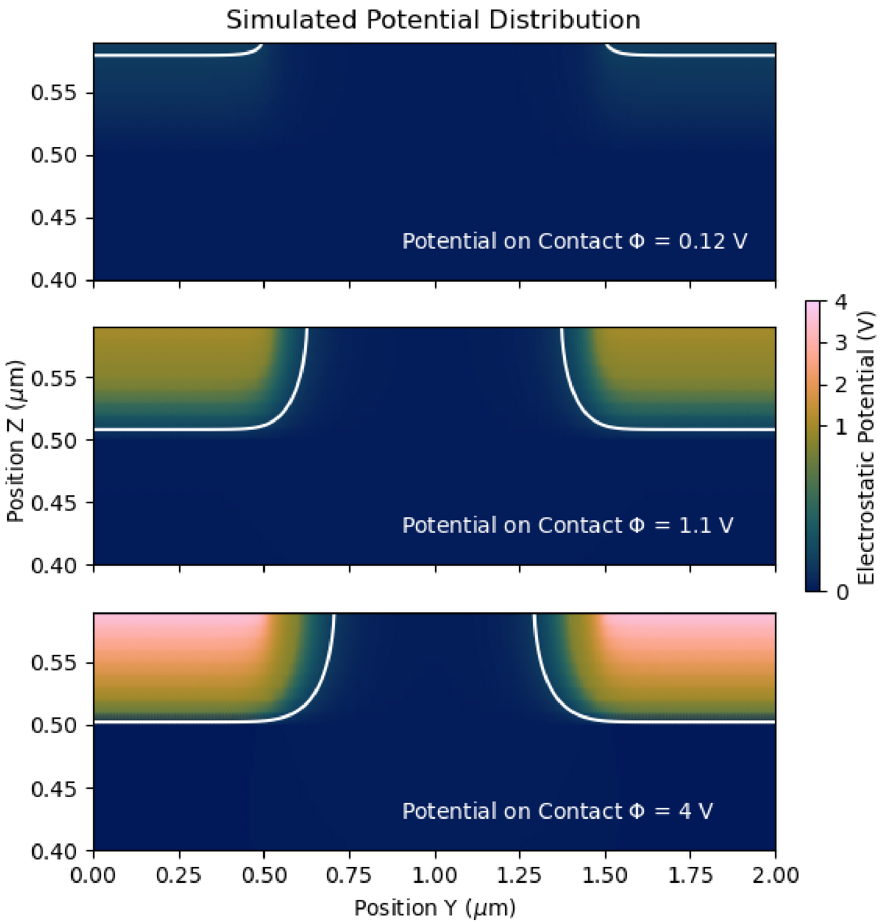

Figure S3: The 2D simulated electric potential distribution with varying applied potential biases on the contact surfaces, as reported in the figure subsets.

be different from the simulated one due to electrostatic accumulation in the actual sample that are hard to model. Furthermore the thickness of the layer of insulating alumina and change in work function between Cr, Ti, and Au were not precisely modeled for simplicity. The representative values are reported in Figure S3 and are at potentials of $\Phi = 0.12\,\mathrm{V}$, $\Phi = 1.1\,\mathrm{V}$, and $\Phi = 4\,\mathrm{V}$.

The resulting effective potential that interacts with the electrons confined to the 2DEG is studied and plotted in Figure S4a. This potential will affect the electron density, following a Thomas Fermi distribution:

$$n_e = \frac{e\,m^*}{\pi\,\hbar^2} \cdot \Phi_{\mathrm{eff}}\,\frac{1}{\mathrm{cm}^2} \tag{1}$$

Where Φ_{eff} describes the effective electric potential at the position of the 2DEG, computed via the simulation. This distribution is plotted in Figure S4b, with changes in the density by 1% indicated by the shaded region. The width of these density changes correspond approximately to the widths obtained via the Transmission Spectrum simulations: $d_{0.12} = 0.93\,\mu\mathrm{m}$, $d_{1.1} = 0.63\,\mu\mathrm{m}$, and $d_4 = 0.48\,\mu\mathrm{m}$.

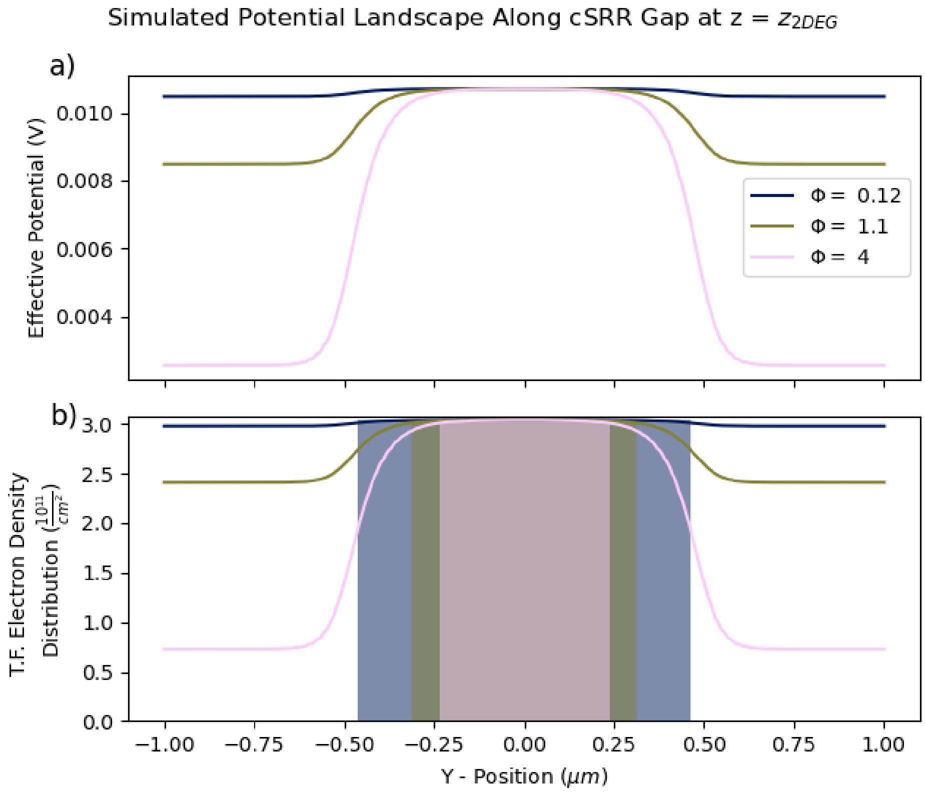

Figure S4: a) The simulated electric potential along the 2DEG channel on a line perpendicular to the gap. b) The computed electron density with shaded regions according to when the density drops by 1% of its maximum value.

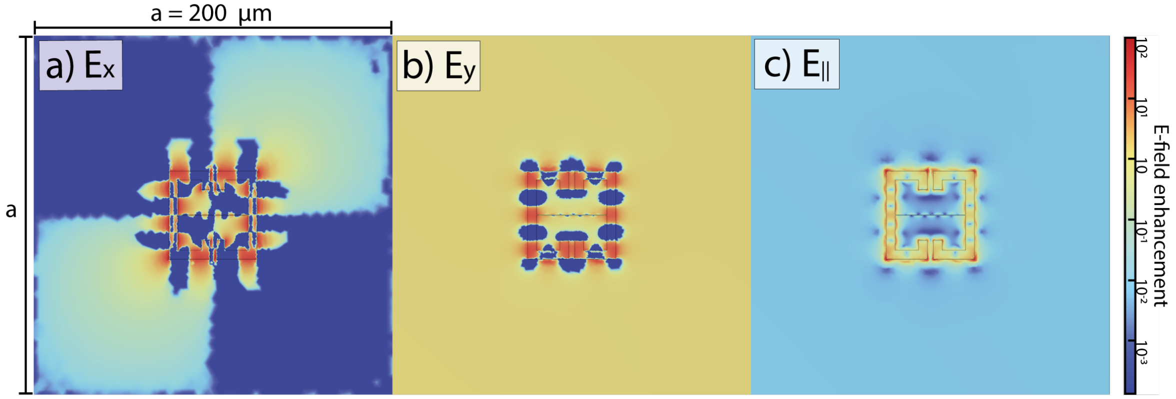

Figure S5: Simulated x-, y-, and in plane components of the electric field inside the 2DEG spatially confined to the cSRR dimensions with a gap size of d $\approx 0.4\mu$m. The plot data is normalized to the incident electric field. The extent of the simulation box is quadratic with sidelength a $= 200\,\mu$m.

Transmission Spectra

The transmission spectra, as well as the simulation of the 2DEG confined to the cSRR region, shown in Figures 3 and 4 of the main text, are performed with the RF-Module. To analyze the transmission, a port is defined with an incident electric field polarized along the y-axis on top of the simulated structure, with a detection port on the back. The structure is simulated with separate periodic boundary conditions in the x-, and y-directions. The simulation was initialized to resemble the resulting sample as closely as possible, with the following material assignments: a silicon material region of t $= 30\,\mu$m to imitate the solid immersion lens on the top, a polymer with t $= 2\,\mu$m to represent the BCB buffer layer, Transition Boundary Conditions to facilitate the computation of the thin cSRR structure and the 2DEG, at 90 nm difference, and a GaAs substrate with t $= 30\,\mu$m. The dielectric function of the 2DEG has been customized to behave as a gyrotropic medium with the permittivity tensor defines as the following:

$$\varepsilon = \begin{pmatrix} \varepsilon_{GaAs} - \dfrac{w_{\text{plas}}^2\,(\omega - i\nu_{\text{coll}})}{\omega\left[(\omega - i\nu_{\text{coll}})^2 - \omega w_{\text{cyc}}^2\right]} & -i\,\dfrac{w_{\text{plas}}^2 w_{\text{cyc}}}{\omega\left[(\omega - i\nu_{\text{coll}})^2 - \omega w_{\text{cyc}}^2\right]} & 0 \\ i\,\dfrac{w_{\text{plas}}^2 w_{\text{cyc}}}{\omega\left[(\omega - i\nu_{\text{coll}})^2 - \omega w_{\text{cyc}}^2\right]} & \varepsilon_{GaAs} - \dfrac{w_{\text{plas}}^2\,(\omega - i\nu_{\text{coll}})}{\omega\left[(\omega - i\nu_{\text{coll}})^2 - \omega w_{\text{cyc}}^2\right]} & 0 \\ 0 & 0 & 1 \end{pmatrix} \tag{2}$$

with the characteristic collision frequency $\nu_{coll} = \frac{e}{m^* \mu}$, depending on the electron effective mass and mobility. The plasma and cyclotron frequencies are equal to the ones reported in the main manuscript.

This type of simulation has been studied for stability against a change in the size of the unit cell to ensure that the presented standing wave pattern within the shaped 2DEG is irrespective of the cell boundaries. An exemplary simulation with unit cell extents of a $= 200\,\mu$m is shown in Figure S5. This simulation shows the same qualitative behavior as the simulation shown in the main body of the manuscript.

We have furthermore performed a simulation to test how the spectrum would change in case of a gate bias with opposite polarity. This would increase the electron density underneath the resonator plane, with the doped region protruding into the cSRR geometry in a similar fashion as the depleted region. In figure S6 we report the resulting spectral map, which shows an increase in coupling strength to roughly $\eta \approx 0.5$ with a cavity asymptote red-shifted by about 25 GHz. The upper polariton remains broadened, owing its non-locality to the static dimensions of the resonator.

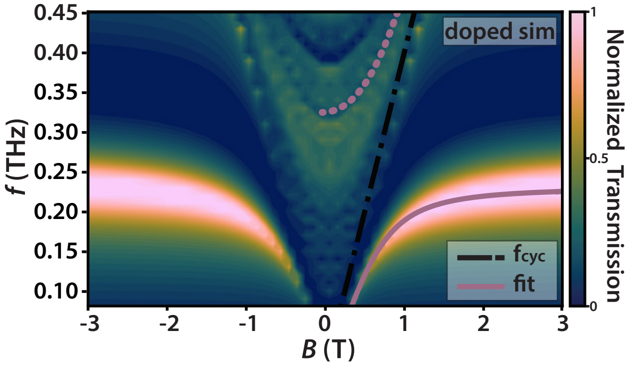

Figure S6: Simulation results for a doping scheme, with an overlaid fit of polariton branches following a

Overlap Factor Simulations

To precisely calculate the overlap factor, the in-plane electric field was exported from the FEM simulations and integrated along the surfaces defined in equation Eq. 5 of the main text. The resulting electric field distributions in the $y - z$ plane are plotted for the varying confinement strengths of the 2DEG in Figure S7. The corresponding simulation results in the $x - y$ plane are given Figure S8. It is evident, that the electric field is expelled from the confined 2DEG. The overlap factors are obtained by numerically integrating the shown distribution, as described in the main manuscript.

Calculation of Number of Electrons

The error in the number of electrons calculated for the interaction in the ungated measurement is given by the respective estimations of the anticrossing magnetic field B and the effective cavity surface S_{eff}. The anticrossing field was assumed to be B = 0.8 T in the calculation performed in the manuscript. Simulations of the empty cavity show a resonance frequency of 270 GHz. However, from the Hopfield fitting procedure of the lower polariton, we can infer a cavity frequency as low as 255 GHz in the coupled system. This yields an estimate for the cavity frequency with the added uncertainty 262.5 GHz ±2.86%. To calculate the anticrossing magnetic field, we must now furthermore estimate the effective mass of the 2DEG. The slope of the observed cyclotron absorption befits an effective mass of $m^* = m_e \cdot 0.07 \pm 1.43\%$.

Collectively, these data then suggest an anticrossing magnetic field varying from 0.629 T to 0.685 T, for a total error of

$$B = 0.657\,\mathrm{T} \pm 4.29\%$$

The effective cavity surface is extracted from finite element simulations of the sample, and in reality will vary with the actual geometry of the resonator lithography. Furthermore, additional layers in the resonator mode (such as the thin layer of alumina), and the actual depth of the 2DEG will affect the broadening of the resonator mode at the position of the 2DEG. Within a standard lithographic process using an invertible resist, we can assume a sidewall accuracy of 50 nm, which results in a 5% uncertainty of the cSRR gap dimensions. Thus allowing for a deviation of up to 5% in the mode surface, we will obtain an electron number of

$$N_{\mathrm{e}}^0 = \frac{eB}{h} \times S_{\mathrm{eff}} \approx 6510 \pm 7\% \tag{3}$$

For the decrease in electron numbers calculated via the coupling strengths, the fit errors offer insights into our estimation error. The RMS errors (normalized to the cavity frequency) of the normalized coupling strengths η_V obtained from the fits are $\mathrm{RMS}_{0V} = 0.00144$, $\mathrm{RMS}_{0.75V} = 0.00147$, $\mathrm{RMS}_{1V} = 0.001676$, $\mathrm{RMS}_{4V} = 0.001708$ for the respective measurements. These errors encompass the deviation of every data point included in the analysis from the theoretical best fit. Observing the χ^2 curvature of the RMS fits around their minimum value, the uncertainty in the coupling strength is of the order of the RMS error itself, yielding a rough maximum estimate of 2% deviation in the fitted coupling strengths, and likewise their

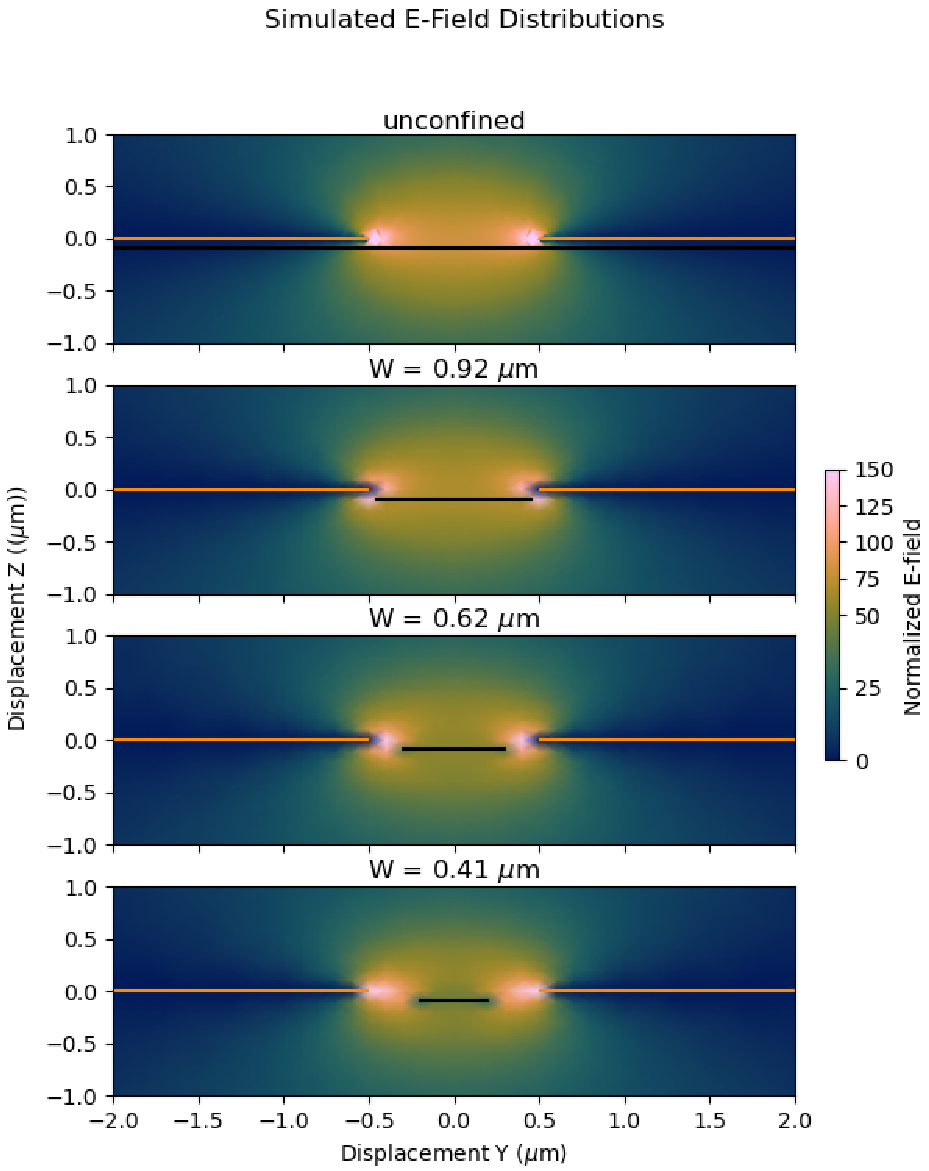

Figure S7: Results from the electric field distribution simulations in the $y - z$ plane for varying confinement strengths of the 2DEG and resulting overlap factors. The extents of the cSRR are overlaid as orange lines, and the extent of the 2DEG are indicated with black lines.

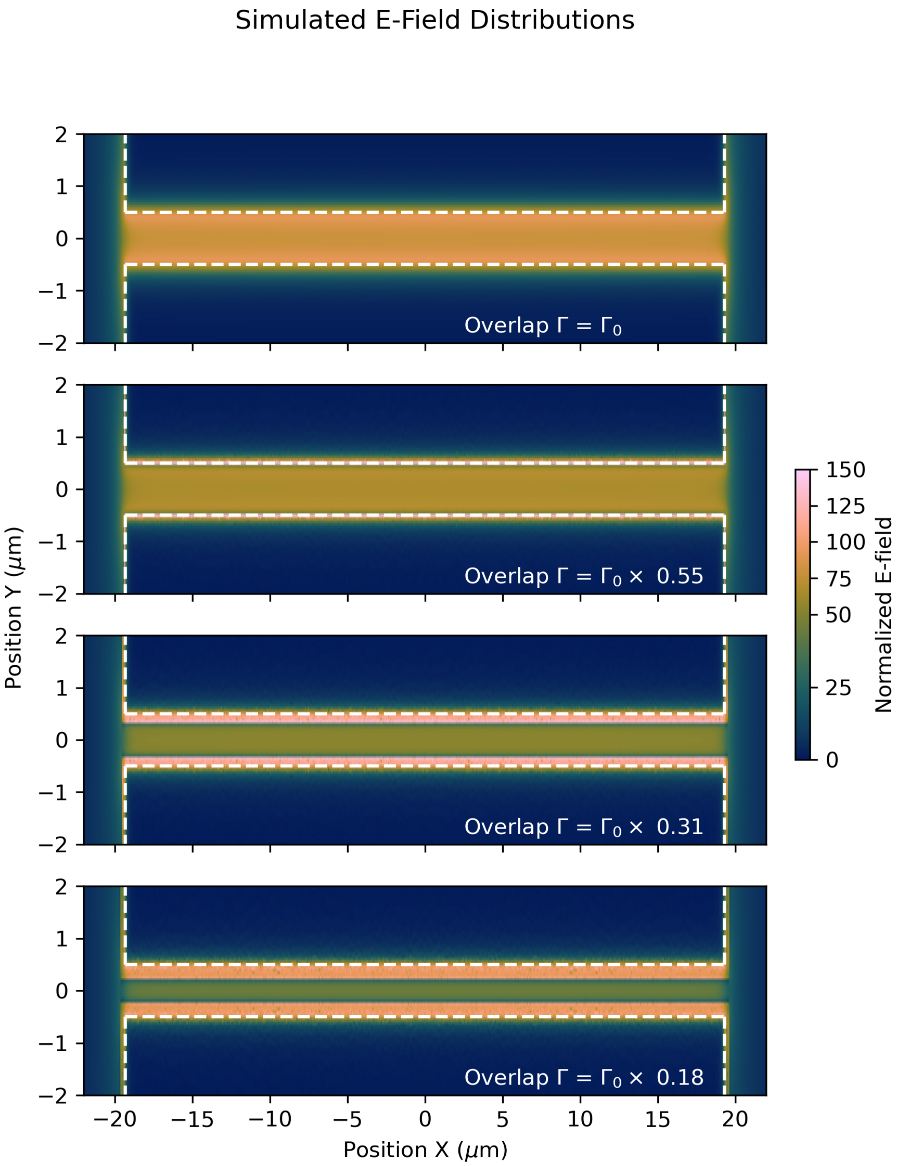

Figure S8: Results from the electric field distribution simulations in the $x-y$ plane for varying confinement strengths of the 2DEG and resulting overlap factors. The fixed extents of the cSRR are overlaid as white dashed lines.

squared ratios:

$$\Delta\frac{\eta}{\eta_0} \approx 2\% \tag{4}$$

The geometric overlap factor obtained from the simulated mode surfaces for the confined electron channels suffer from the same uncertainty as the original estimate of S_{eff}. Additionally to the realistic resonator geometry uncertainty, the electron channel widths are obtained by fitting the simulated spectra to the measured spectra. When the simulations were performed, the electron channel widths were swept in a parametric sweep, allowing for increments of $0.01\,\mu$m and subsequently compared to the experimental results. The best fits therefore leave room for an error in the channel width of around $\Delta d = 0.005\,\mu$m. To propagate this error to the overlap factors, we can assume a constant in-plane electric field distribution within the 2DEG and multiply by the added width of the electron channel. This argument is valid given that the E-field is largely expelled from the 2DEG, as becomes visible from the sidecut views of the simulated field distributions (provided Figure S7). This yields a total error in the overlap factors of approximately

$$\Delta\Gamma_V \approx 2\% \tag{5}$$

Taking all of these errors into account, the overall uncertainty in electron number in our calculations is approximately the same for both methods, and maximally 10% of the given values.
